# The glass transition in high-density amorphous ice

**DOI:** 10.1016/j.jnoncrysol.2014.09.003

**Published:** 2015-01-01

**Authors:** Thomas Loerting, Violeta Fuentes-Landete, Philip H. Handle, Markus Seidl, Katrin Amann-Winkel, Catalin Gainaru, Roland Böhmer

**Affiliations:** a Institute of Physical Chemistry, University of Innsbruck, Innrain 80-82, A-6020 Innsbruck, Austria; b Fakultät Physik, Technische Universität Dortmund, Otto-Hahn-Straße 4, D-44221 Dortmund, Germany

**Keywords:** Glass transition, High-density amorphous ice, Dielectric relaxation spectroscopy, Differential scanning calorimetry, Polyamorphism

## Abstract

There has been a long controversy regarding the glass transition in low-density amorphous ice (LDA). The central question is whether or not it transforms to an ultraviscous liquid state above 136 K at ambient pressure prior to crystallization. Currently, the most widespread interpretation of the experimental findings is in terms of a transformation to a superstrong liquid above 136 K. In the last decade some work has also been devoted to the study of the glass transition in high-density amorphous ice (HDA) which is in the focus of the present review. At ambient pressure HDA is metastable against both ice I and LDA, whereas at > 0.2 GPa HDA is no longer metastable against LDA, but merely against high-pressure forms of crystalline ice. The first experimental observation interpreted as the glass transition of HDA was made using in situ methods by Mishima, who reported a glass transition temperature T_g_ of 160 K at 0.40 GPa. Soon thereafter Andersson and Inaba reported a much lower glass transition temperature of 122 K at 1.0 GPa. Based on the pressure dependence of HDA's T_g_ measured in Innsbruck, we suggest that they were in fact probing the distinct glass transition of very high-density amorphous ice (VHDA). Very recently the glass transition in HDA was also observed at ambient pressure at 116 K. That is, LDA and HDA show two distinct glass transitions, clearly separated by about 20 K at ambient pressure. In summary, this suggests that three glass transition lines can be defined in the p–T plane for LDA, HDA, and VHDA.

## Highlights

• The recent literature about the glass transition in amorphous ices is reviewed.
• LDA, HDA and VHDA show three distinct glass transition temperatures T_g_.
• HDA's T_g_ at 1 bar is 20 K lower than LDA's T_g_.
• The pressure dependence for HDA's T_g_ is: T_g_(p) = 115.9 K ∗ (1 + p / 0.00779 GPa)^0.056^.
• Calorimetry and dielectric data are consistent with liquid nature above T_g_.

## 1 Introduction

Amorphous ices are the dominant form of water in the universe, even though they do not naturally form on Earth's lithosphere. They may occasionally form at < 150 K in the coldest region of Earth's atmosphere, near the mesopause at altitudes of about 80 km [1]. The most widespread occurrence of amorphous ice is on interstellar dust, in comets, and many other astrophysical environments including the Saturnian rings [2]. The conditions of the formation of amorphous ices in nature vary greatly. Crystalline ice may amorphize under the influence of UV- or ion-irradiation [3], [4]. On interstellar dust particles, amorphous solid water may form by chemical vapor deposition at 10 K, involving reaction of O, H, O_2_, H_2_ and OH, or by direct water vapor deposition onto dust particles. Also in the laboratory many routes to amorphous ices exist. Some of them are: a) deposition of water vapor onto cold substrates [5], b) ultrafast cooling of liquid water droplets [6], c) pressure-induced amorphization of crystalline ice [7], [8], and d) temperature- or pressure-induced amorphous–amorphous transformations [9], [10], [11], [12]. One of the main questions related to amorphous ices is the question whether or not they are thermodynamically continuously connected with liquid states. If they were, then the amorphous ices would need to be regarded as vitrified liquids, i.e., glassy states. Alternatively the amorphous ices could be regarded as distorted crystalline phases, nano-crystals, or crystal-like states. A thermodynamic connection with the stable liquid at ambient temperature is very difficult to check, if not impossible, because amorphous ices crystallize very rapidly above the crystallization temperature T_X_. Depending on pressure T_X_ is about 140–190 K [13], [14], [15], [16], [17], [18], [19], [20], [21], [22]. While the liquid can be supercooled rather easily below the melting temperature it crystallizes very rapidly upon cooling below the homogeneous nucleation temperature T_H_. Depending on pressure T_H_ is about 181–235 K [23]. That is, there is a gap of about 30–40 K width, in which crystal nucleation and growth are very rapid and can hardly be avoided. This gap, in which non-crystalline water cannot be observed on time scales exceeding 1 s, has become known as “no-man's land”. In very recent studies, using ultrafast probing of evaporatively cooled droplets, some unfrozen, liquid droplets could be investigated at temperatures down to 227 K at (sub-)ambient pressure after flight times of a few milliseconds [24]. The crystallization of ice can even be avoided altogether by ultrarapid cooling at rates ≥ 10^7^ K/s, whereas at cooling rates of 10^5^ K/s a significant fraction of the droplets crystallizes [25]. After ultrafast cooling the droplets are in a vitrified liquid, glassy state called hyperquenched glassy water (HGW). By contrast to the very recent study [24], in which in situ probing down to 227 K was possible, the droplets could not be studied in situ upon cooling in the “no-man's land”, e.g., at 190 K for the hyperquenching experiment. Instead only the deposits typically stored at 77 K could be investigated [25]. Since HGW is produced by cooling stable liquid droplets fast enough to avoid crystallization it seems that there has to be a thermodynamic connection between HGW and stable liquid droplets [6], [26], [27]. The question whether quantities such as heat capacity show maxima or singularities upon cooling [28], [29], [30] is still unresolved, though. For amorphous ices produced via other routes the thermodynamic connection was even completely unclear until recently. This is especially true for amorphous ices which require pressure-induced amorphization of crystalline ices in the course of their preparation, such as high-density amorphous ice (HDA) [7], [8]. However, the connection between the liquid and the amorphous state cannot be probed only upon cooling the liquid, but also upon heating the amorphous solid. Ultrafast heating and simultaneous ultrafast probing would be required to beat crystallization and to check for thermodynamic continuity within the no-man's land. This has been out of reach in research on amorphous ices so far even though calorimetry methods reaching heating rates on the order of 10^5^ K/s were employed to study them [31], [32]. However, it has been possible to study whether or not a glass transition can be observed upon heating amorphous ice prior to crystallization and/or prior to the transformation to other amorphous ices [20], [31], [32], [33], [34], [35], [36], [37], [38], [39], [40], [41], [42], [43], [44]. This glass transition would lead to an ultraviscous liquid state which is also called deeply supercooled liquid water. If the glass transition was absent amorphous ice may either be crystal-like or glassy, but does not reach its liquid state prior to transformation. For HDA a crystal-like nature was suggested on the basis of inelastic neutron and X-ray scattering experiments indicating similarity to high-pressure ice polymorphs such as ice VI or ice IX [45], [46], [47], [48], [49], [50].

Amorphous ices, glassy water, and (deeply) supercooled liquid water have been subject of several reviews in the last decade [28], [51], [52], [53], [54], [55]. These reviews have touched on the question regarding the glass transition in low-density amorphous ices. The present work expands on these reviews by focusing on the recent advances related to the possibility of a glass transition in HDA.

## 2 Ice polyamorphism

In terms of densities the amorphous ices can be grouped into three categories: low-density amorphous ices (LDAs), high-density amorphous ices (HDAs), and very-high density amorphous ices (VHDAs) [54]. As a function of pressure three linear regimes of amorphous ice bulk densities can be identified at temperatures just below crystallization [54]. The ambient pressure densities (at ~ 80 K) of the three amorphous ices are 0.93 ± 0.02, 1.15 ± 0.02, and 1.26 ± 0.02 g/cm^3^, respectively [56]. Especially after vapor deposition at low temperatures amorphous ices may be microporous [57] and show specific surface areas of up to 2700 m^2^/g [58]. Before annealing some of these ices may contain a large number of micropores, and show porosities of up to 70%. After annealing at temperatures > 120 K the micropores collapse and compact amorphous ices result [59]. The presence of empty pores reduces the overall densities significantly, while bulk densities are not affected. Amorphous ices of bulk ambient pressure densities < 0.90 g/cm^3^ and > 1.30 g/cm^3^ have not been identified so far. Also amorphous ices of densities of 0.96–1.12 g/cm^3^ are unknown. This gap in densities is a clear indication of what has become known as polyamorphism [60], [61], [62] — the occurrence of (at least two) amorphous forms of ice [9]. Furthermore, the polyamorphic ices differ significantly according to structural methods such as X-ray or neutron diffraction. All of them show a hydrogen-bonded network with tetrahedral coordination of water molecules. In particular, the number of interstitial water molecules can be employed to categorize the amorphous ices. LDA does not show any interstitial water molecules, whereas HDA and VHDA show one and two interstitials, respectively [63], [64], [65]. These interstitial molecules are located at OO-distances of 3.0–3.4 Å from a (randomly chosen) central water molecule. They are not bonded to the central water molecule by hydrogen bonds, which are characterized by OO-distances of 2.77–2.85 Å [10], but are instead located in between the first and second coordination shells. Just like any water molecule in amorphous ices the interstitial water molecules themselves are tetrahedrally coordinated so that the Walrafen water pentamer can be regarded as the basic building block of all amorphous ices [66].

The term polyamorphism [60], [61], [62] was used in different ways in literature. It has been used not only in the correct sense, but also in flawed ways. “False polyamorphism” involves two (or more) amorphous states differing just in terms of the degree of relaxation. Among these amorphous states there are more stable and less stable ones. The less stable states relax continuously towards the more stable states, whereas a relaxation from the more stable to the less stable state will never be observed. That is, the relaxation is irreversible and only proceeds one way (monotropic). “False polyamorphism” is the same as relaxation. By contrast, “true polyamorphism” is understood to involve changes in topology, e.g., connectivity or the number of interstitial water molecules. “True polyamorphism” also necessitates that conditions can be found at which the polyamorphic states are at metastable equilibrium. That is, there is reversibility and the transitions may proceed in both directions with hysteresis (enantiotropic). For the amorphous ices, it is possible to switch back and forth between the three polyamorphic states by isothermal compression and decompression experiments [12], [13], [67], [68]. In the case of the LDA ↔ HDA transition a very sharp transition involving a sudden 25% change in density is observed as a function of pressure, in spite of the low transformation temperature of 130 K [13]. The upstroke and downstroke transitions show hysteresis, with an equilibrium pressure of about 0.20 GPa; below which LDA is the most stable amorphous phase and above which HDA is the most stable amorphous phase [69], [70]. Also in the case of HDA ↔ VHDA isothermal upstroke and downstroke transitions can be observed, with an equilibrium pressure of about 0.75 GPa [12], [67]. Below 0.75 GPa HDA is the most stable amorphous phase, and above 0.75 GPa VHDA is the most stable amorphous phase. By contrast to the very sudden and sharp LDA ↔ HDA transition, the HDA ↔ VHDA transition is less sharp and involves a 10% density change smeared over a ≈ 0.2 GPa wide pressure interval. While we regard these experimental observations sufficiently meet the criteria for “true” polyamorphism, it was suggested from computer simulations of water models that VHDA may instead be a relaxed form of HDA [71], [72], [73].

Recent molecular dynamics simulations on ST2 water and the monatomic Fermi–Jagla potential have paved an experimentally tractable way of distinguishing true from false polyamorphism [74], [75], [76], [77], [78]. Both models show (true) polyamorphism in the glassy state and liquid–liquid separation at higher temperatures. The two amorphous states (LDA and HDA) in these models show two distinct glass-to-liquid transition temperatures T_g_. That is, the observation of two well separated glass transition temperatures at a specific pressure indicates true polyamorphism. In the pressure–temperature plane two T_g_ lines, T_g,1_ and T_g,2_, can be defined. One of these lines pertains to the most stable amorphous/liquid state, and the other to an amorphous/liquid state metastable against the other amorphous/liquid state. The T_g_ line pertaining to the most stable amorphous/liquid state shows a discontinuity at the liquid–liquid coexistence line where there is a switch of the most stable amorphous state, and thus a switch from T_g,1_ to T_g,2_ [79]. By contrast, for other models such as SPC/E water, which do not show (true) polyamorphism the glass transition temperatures for all amorphous states coincide [74]. In such simulations the glass-to-liquid transition can be recognized by a sudden increase in mean-square displacement of oxygen atoms, a step-like increase in heat capacity or thermal expansivity or a kink in volume vs. temperature curves [74], [80], [81], [82], [83].

Co-existence of two amorphous ices was also found in other simulations [84], in agreement with experiments [85], [86]. Instead of reaching two glass transition temperatures, both amorphous ices connect with the same ergodic liquid, resulting in an amorphous–amorphous–liquid triple point in the non-equilibrium phase-diagram [84]. However, it is hard to reconcile the two well separated glass transitions recognized in the experiments summarized in the following with the scenario proposed in [84]. From experiments there is no hint for a triple point and a co-existence of two amorphous ices and one liquid, but support for the two-liquid scenario. However, the liquid–liquid critical point proposed for two-liquid models is neither confirmed nor refuted by the experimental observation of two glass transitions, because this liquid–liquid critical point is “virtual”. Both ultraviscous liquids crystallize rapidly at > 160 K, and so the possibility of a second critical point at > 200 K cannot be probed using experiments with sensitivity on the time scale of minutes or hours. Even if technology was available to beat the time scales of crystallization, one could not observe critical singularities associated with a metastable liquid–liquid phase separation very close to the critical point. This is because the critical slowing down accompanying the approach of this point necessitates observation times longer than the crystallization time [87]. Nevertheless, Mishima has interpreted his experimental results on the melting curves of high-pressure ice phases to be signatures for a liquid–liquid critical point near 0.1 GPa and 220 K [88].

**Scheme 1:**
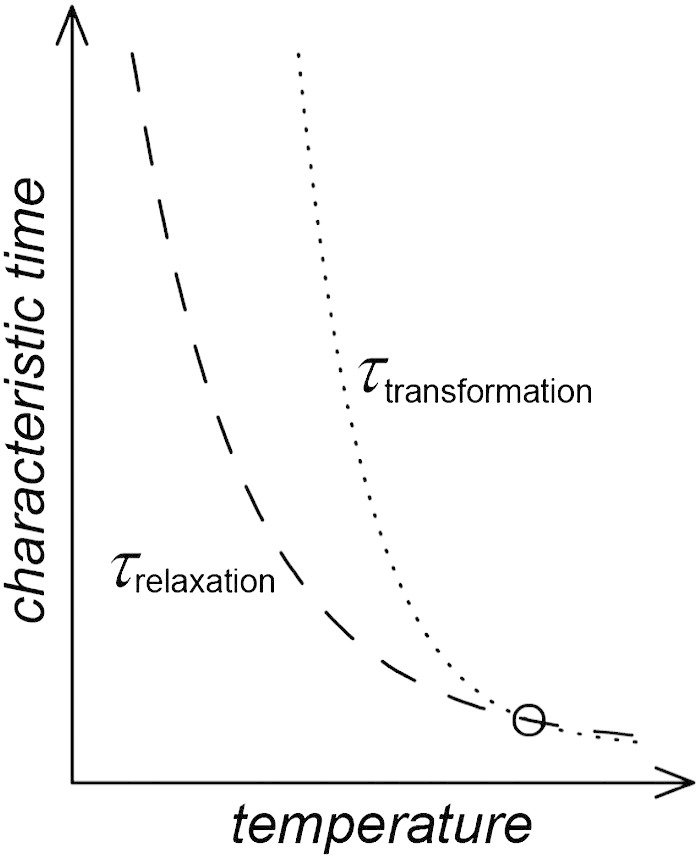
Sketch of transformation times τ_transformation_ and structural relaxation times τ_relaxation_ of eHDA. For this type of HDA, structural relaxation is faster than transformation, provided that the temperature is kept low enough.

Amorphous ices are by definition non-equilibrium states of matter, which slowly relax towards equilibrium. The state of lowest Gibbs free energy is of course crystalline, e.g., ice XI below 72 K and hexagonal ice above 72 K at ambient pressure. By keeping the temperature low enough not only to avoid transformation to crystalline material, but also to avoid amorphous–amorphous transformations it is possible to relax amorphous ices, which are metastable both with respect to another amorphous ice and to crystalline ice polymorphs. That is, there has to be a separation between time scales of crystallization and of relaxation. More specifically, for a state to be observable the time scale for transformation (to more stable crystalline or amorphous material) has to be much longer than the time scale for reaching the metastable equilibrium, as indicated in Scheme 1. When HDA was reported for the first time it was prepared by pressurizing hexagonal ice beyond 1.2 GPa at 77 K [7]. This form of HDA is nowadays referred to as unrelaxed HDA (uHDA) and displays rather fast transformation times, comparable to the relaxation times [89]. By annealing HDA at 0.1–0.2 GPa Nelmes et al. [11] and Handle et al. [90] were able to obtain a more relaxed form of HDA called expanded HDA (eHDA). Similarly, Winkel et al. obtained the eHDA state by decompression of VHDA at 140 K [86]. The time scales for transformation are much longer in eHDA than in uHDA [89]. eHDA can be kept for many years at 77 K, for hours at 123 K, and for minutes at 130 K without noticeable transformation to LDA at ambient pressure [89]. Thus, relaxation, equilibration, and the glass transition of HDA can be studied at T < 130 K at ambient pressure even though LDA is more stable than HDA at this pressure [44].

## 3 Glass-to-liquid transition in high-density amorphous ice?

The central question concerning the amorphous ices is whether they are thermodynamically continuously connected with the deeply supercooled liquids usually associated with them or not. In other words, the question is whether or not the amorphous ices are glasses connected to these liquids by a glass-to-liquid transition. In the case of LDA a glass transition was reported to occur at 136 ± 2 K using calorimetry at heating rates of 10 K/min. The ambient pressure glass transition in LDA is observed at this temperature no matter whether the LDA was obtained by the route of vapor deposition [36], vitrification of micron-sized droplets [34], or after pressure-amorphization of ice I [41]. This glass transition is very feeble and associated with an increase in heat capacity of ΔC_p_ ~ 1 J K^− 1^ mol^− 1^. This is much less than the heat capacity increase observed at the glass transition of most other liquids. In fact, it is so small that it was overlooked even by experienced researchers. There is an ongoing scientific controversy about the question whether the increase in heat capacity is linked to an increase in translational mobility of water molecules or whether it is linked to increased mobility of H atoms only [91]. The former is related to a softening of the sample and change in viscosity, i.e., a transformation to the liquid state. The latter is referred to as orientational glass transition and not associated with a transformation to the liquid state, but rather from one solid state, in which molecular rotations are immobilized to another solid state, in which rotations are possible. Orientational glass transitions can also be found in crystals, e.g., in ice I_h_ [92] or in ice XII [93]. We refer the interested reader to recent reviews about the topic of the controversial glass transition in LDA, e.g., chapter X in [55] or chapter III.E in [94]. Irrespective of the nature of this glass transition most of the experimental data indicate that low-density water just above 136 K is not fragile, but strong in terms of Angell's classification [40], [95], [96], [97], [98], [99], [100].

### 3.1 Experiments at high pressure

In the present article the focus is on experimental studies of the glass transition in HDA. While HDA was discovered and the concept of polyamorphism in water was invented about thirty years ago [9], the question regarding the glass transition in HDA remained elusive for a while. The first experiments suggesting a connection between HDA and the pressurized liquid state were published in 2001 by Mishima and Suzuki [101]. These authors succeeded in pressurizing emulsified liquid water to ~ 0.5 GPa, cooling it at 10^3^–10^4^ K/s, and obtaining a vitrified, glassy state. This vitrified state experiences a sudden volume expansion and converts to LDA when heating to ~ 130 K at ambient pressure. That is, the pressurized liquid transforms to HDA upon cooling, just like the ambient pressure liquid transforms to LDA upon hyperquenching [25]. The pressurized liquid transforms more easily to the glassy state than the ambient pressure liquid: The study of Mishima and Suzuki demonstrates that the cooling rates required to avoid crystallization can be about 3 orders of magnitude smaller than the 10^7^ K/s that are required at ambient pressure. The relation between HDA and the pressurized liquid also becomes clear when examining the ice phases crystallizing from them. Upon cooling the pressurized liquid slowly, it may transform either to ice IV [102], [103], [104] or to ice XII [105]. Both of these ice phases form as metastable phases within the stability fields of ice V and of ice VI. Similarly, upon heating HDA the same two metastable phases crystallize: ice IV at small heating rates [106], [107] and ice XII at large heating rates [108], [109] or under shockwave heating [110], [111]. These experiments clearly suggest a large degree of similarity between the pressurized liquid and HDA and underscore the connection between them. However, these experiments do not answer the question about the glass transition temperature, i.e., whether or not the glass transition precedes crystallization upon heating HDA.

**Fig. 1:**
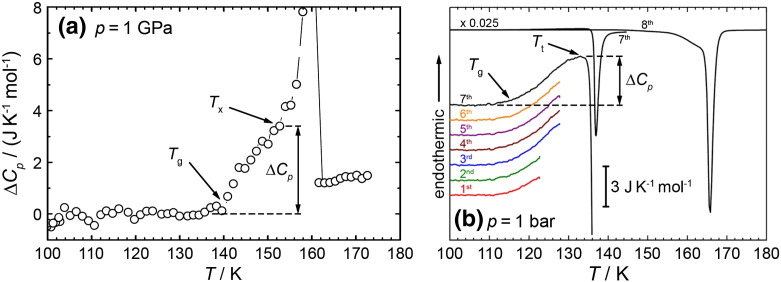
Relative changes in heat capacity ΔC_p_ for (as we argue) VHDA samples on heating at 1.0 GPa (a) and HDA samples on heating at ambient pressure (b). T_g_ represents the onset of the glass transition, and T_X_ and T_t_ represent crystallization to high-pressure polymorphs and transformation to LDA, respectively. (a) is adapted from reference [117] and (b) was measured analogously to the DSC data shown in [44], except that more heating/cooling cycles were conducted. The sample was measured eight times upon heating: twice to 123 K, four times to 128 K, once to 143 K (resulting in transformation to LDA), and finally to room temperature (successively resulting in crystallization of LDA and melting of ice I).

**Fig. 2:**
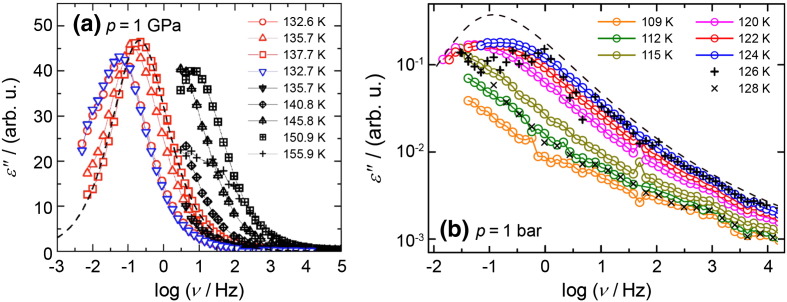
Dielectric loss ε″ for VHDA at 1.0 GPa (a) and HDA at ambient pressure (b) and temperatures as indicated. The dashed line in panel (a) represents a fit to the data, while the dashed line in panel (b) represents data of supercooled glycerol [138]. Please note that the peaks appear sharp in panel (a), but broad in panel (b) because the y-axis in panel (a) is linear, whereas it is logarithmic in panel (b).

Adapted from [115], [44], respectively.

**Fig. 3:**
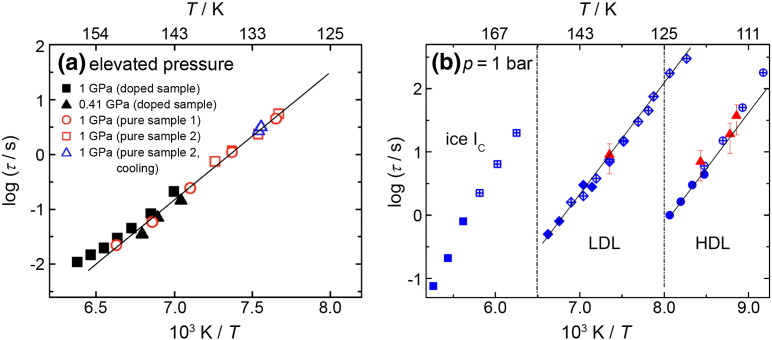
Dielectric relaxation times τ_dielec_ for VHDA at 0.41 and 1.0 GPa (a) and for HDA ( and ), LDA ( and ), and ice I_c_ ( and ) at ambient pressure (b). Solid lines represent Arrhenius fits. In (b) solid symbols were read from the dielectric loss peak, open-crossed symbols were determined by time temperature superposition and  represents calorimetric relaxation times estimated from experiments at different heating rates and Hodge's formula [139].

Adapted from [115], [44], respectively.

The main techniques employed for investigating the glass transition are calorimetry and dielectric relaxation spectroscopy, both at ambient pressure and at high-pressure conditions. Such data collected on amorphous ices are shown in Fig. 1, Fig. 2, Fig. 3. The glass transition in HDA can be checked for at ambient pressure because the transformation to the low-density phase at ambient pressure is hindered by a substantial energy barrier and proceeds in a jump-like manner only above a specific temperature. At 1 bar HDA is metastable with respect to LDA, which itself is metastable with respect to ice I_c_, ice I_h_, and ice XI. In the case of equilibrated HDA the thermal energy available at temperatures of < 130 K is not sufficient to achieve the transformation within an hour. This is in contrast to other pressure-densified materials, which do not show polyamorphism. In such materials, e.g., polymers, the pressure-densified state continuously progresses to lower densities upon heating at ambient pressure [112]. The small barriers against the transformation to lower densities can easily be surmounted by providing thermal energy.

**Fig. 4:**
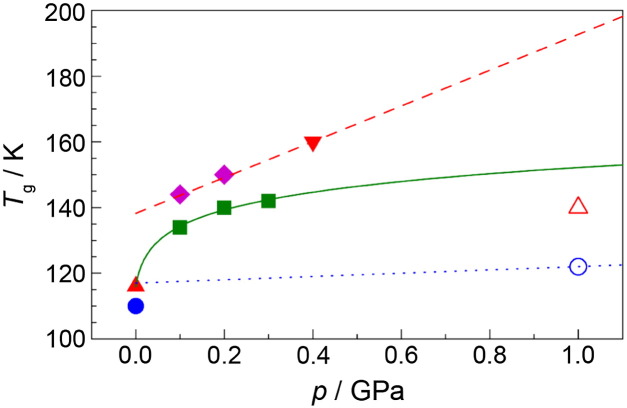
Summary of glass transition temperatures T_g_ reported in literature for HDA (filled symbols, red dashed line and green solid line) and VHDA (open symbols, blue dotted line) in literature as a function of pressure p. HDA: Dielectric relaxation spectroscopy at ~ 0.01 K/min () [44], differential scanning calorimetry at 10 K/min () [44], volumetry at 2 K/min () [20], and isothermal–isobaric anneal experiments () [90]. The red dashed line represents an extrapolation from measurements of thermal effects in emulsified HDA samples in the presence of LiCl () [113]. The green line represents an Avramov fit [126] to the volumetric data points () and the calorimetric point () and is given by T_g_(p) = 115.9 K × (1 + p / 0.00779GPa)^0.056^. VHDA: in situ heat capacity measurements () [117] and in situ dielectric spectroscopy () [115]. The blue dotted line indicates the pressure dependence in the dielectric relaxation time of VHDA.

The glass transition temperature of HDA was first estimated by Mishima for emulsified water [113]. He was monitoring temperature changes upon decompression and attributed a weakly endothermic event just prior to the transformation to the glass-to-liquid transition in HDA. This was observed at 160 K and 0.40 GPa ( in Fig. 4). The pressure dependence of the glass transition was measured indirectly in a study of salty HDA solutions, in which a similar endothermic event could be observed in a broad pressure range [113]. These experiments suggest that HDA's glass transition increases from about 140 K near ambient pressure to 190 K at 1 GPa (red dashed line in Fig. 4).

Andersson and Inaba were using in situ dielectric relaxation spectroscopy [114], [115], [116] as well as in situ heat capacity and thermal conductivity measurements [117] to investigate the nature of amorphous ice under pressure. Even though they do not call their sample VHDA, the sample pretreatment done in their studies suggests that VHDA is actually the material under scrutiny. Andersson observed an increase in heat capacity of 3.4 ± 0.2 J K^− 1^ mol^− 1^ upon heating at 1.0 GPa and 140 K (see Fig. 1(a)), prior to crystallization at 153 ± 1 K [117] and attributed this increase to a transformation from the glassy solid to an ultraviscous liquid. After heating HDA at 1.0 GPa to 148 K, i.e., to the ultraviscous liquid state, and recooling, i.e., vitrification, Andersson observed a linear increase in thermal conductivity when heating up to 140 K, a linear decrease for 140–153 K and finally another increase at 153 K. That is, the thermal conductivity of HDA increases with temperature at 1 GPa, whereas it decreases in the case of high-density liquid water (HDL) before crystallization commences at 153 K [117]. These findings imply that VHDA experiences a glass-to-liquid transition on the time scale of seconds at 1.0 GPa. Andersson's T_g_ is lower by 50 K than the T_g_ of 190 K estimated by Mishima for the same pressure [113] (compare red dashed line and  at 1.0 GPa in Fig. 4). Even though both authors call their samples “HDA”, we believe that the reason for the discrepancy is that Mishima was studying HDA, whereas Andersson was studying VHDA.

The dielectric loss spectra obtained by Andersson and Inaba at high-pressure conditions [114], [115], [116] are depicted in Fig. 2(a). A loss peak is detected at about 10^− 1^ Hz for HDA at ~ 138 K and 1 GPa. The corresponding relaxation times as a function of temperature are shown in Fig. 3(a). This figure demonstrates that the relaxation times are barely affected by changing the pressure from 1.0 to 0.41 GPa. We think that also the relaxation times obtained by Andersson and Inaba at 0.41 GPa reflect the relaxation times in VHDA, because VHDA does not transform back to HDA even at 0.41 GPa [86] using the decompression protocol employed by them [115]. Furthermore, they showed that the use of KOH doping, added in order to facilitate reorientational motions of water molecules, does not alter the dielectric relaxation times. This suggests that either reorientational dynamics is not at the origin of the dielectric loss peak or that KOH does not influence reorientational dynamics. The latter seems unlikely because KOH is known to accelerate reorientational dynamics in crystalline ices by several orders of magnitude. In the case of hexagonal ice this increase in reorientational dynamics allows for the transformation to proton-ordered ice XI below 72 K, which is kinetically hindered without KOH doping [92], [118], [119], [120], [121], [122]. One could also assume that KOH doping might be ineffective in amorphous ices if one regards them as states involving a large number of point defects even in the absence of intentional doping. However, there is no evidence for the presence of more point defects in undoped amorphous ices than in undoped crystalline ices [123], [124]. One can, therefore, expect enhancement of reorientational dynamics by the introduction of point defects through doping. In this view, observation of similar relaxation times in doped and undoped ices suggests that the relaxation mechanism is not reorientational dynamics. In fact, this view is corroborated by the dielectric relaxation times referring to reorientational dynamics measured in crystalline ices (see cubic ice data in Fig. 3(b)). Extrapolating the dielectric relaxation times for reorientational dynamics measured for cubic ice in the temperature range of 150–200 K, e.g., to 120 K (cf. Fig. 3(b)) one expects reorientational relaxation times of 10^6.5^ s. These are orders of magnitude slower than the dielectric relaxation times measured for LDA (10^2.8^ s) and HDA (10^0.5^ s) at this temperature and suggest that a mechanism other than water rotation, e.g., translation of water molecules, is operative producing the dielectric loss peaks observed for both LDA and HDA (see Fig. 2).

Seidl et al. [20] and Handle et al. [90] estimated HDA's T_g_ in the pressure range of 0.1–0.3 GPa. Seidl et al. used volumetry combined with X-ray diffraction to investigate the isobaric thermal expansivity of HDA. At T_g_ the volume vs. temperature curve shows a kink, and, thus, thermal expansivity shows a step-like increase. However, such type of behavior can be observed not only as a result of the repeatable relaxation processes underlying a glass-to-liquid transition, but also to other types of relaxation. In particular, non-repeatable relaxation processes related to the release of strain, etc. may also be at the origin of such behavior. In order to discriminate relaxations, which are repeatable or non-repeatable Seidl et al. were heating and recooling HDA up to six times. Furthermore, in this study there was a necessity to find a maximum temperature for the heating cycles in order to exclude volume changes caused by crystallization [22]. For instance, HDA could be heated six times to 144 K at 0.20 GPa without detecting traces of crystallization by X-ray diffraction, whereas five times heating to 145 K at 0.20 GPa has already resulted in partial crystallization (as inferred by the occurrence of sharp Bragg reflections in addition to the broad halo peak originating from HDA) [89]. When heating HDA for the first time after its production (using the protocol established by Winkel et al. [12], [86]) irreversible relaxation was noticeable by volume changes at T < T_g_. After recooling and heating a 2nd time a deviation from linear sample expansion was observed at higher temperatures than in the 1st run. In the 3rd and all subsequent heating runs this deviation was found at the same temperature as in the 2nd run. Also the change in expansivity up to the maximum temperature was observed to be nearly the same in all runs subsequent to the 2nd one. Using X-ray diffraction the absence of noticeable crystallization after six heating runs was confirmed. The temperature characterized by a sudden change in expansivity and the volume relaxation indicated by the kink in the volume vs. temperature curve was assigned to be HDA's glass-to-liquid transition temperature at high-pressure conditions. Using this cumbersome protocol T_g_ of HDA was determined to be 134 ± 2, 140 ± 2, and 142 ± 2 K, at 0.10, 0.20, and 0.30 GPa, respectively [20] ( in Fig. 4).

An alternative way of determining HDA's T_g_ under high-pressure conditions was used by Handle et al. [90] at 0.10 and 0.20 GPa. In their experiments the structural relaxation time of HDA was measured from time-dependent isothermal–isobaric experiments. An HDA sample was produced (following Mishima's protocol [7]), decompressed to 0.10 GPa or 0.20 GPa at 77 K, and then heated to 110, 125, 130, or 135 K. The samples were then left for an anneal-time at a specific combination of pressure and temperature, and then quench-recovered and analyzed by using X-ray diffraction for crystallinity and differential scanning calorimetry for thermal stability against transformation to LDA at 1 bar. The evolution of thermal stability of the amorphous sample with anneal-time could be mapped by repeating the experiment several times for each combination of pressure and temperature, applying different times between 0 and about 10,000 s. The thermal stability increased with increasing anneal-time and approached a limiting plateau value in the case of 125, 130, or 135 K. At 110 K the thermal stability increased with time, but a plateau value was not closely approached even after an anneal-time of a few hours. At higher temperatures the relaxation times could no longer be determined because of the interference of rather fast crystallization. Structural relaxation times τ_relaxation_ could be extracted from the thermal stability vs. anneal-time plots [90]. Relaxation times were found to be slightly smaller at 0.10 GPa than at 0.20 GPa and they decrease with increasing temperature. Furthermore τ_relaxation_ was found to show an Arrhenius behavior (i.e., linear dependence of lnτ_relaxation_ vs. 1/T), with an activation energy of 40 ± 10 kJ/mol at 0.10 GPa and 34 ± 5 kJ/mol at 0.20 GPa. This suggests HDL to be a strong liquid [125]. By extrapolating the relaxation times to higher temperatures T_g_ could be estimated from the condition τ_relaxation_ = 100 s resulting in 144 ± 2 K at 0.10 GPa and 150 ± 12 K at 0.20 GPa ( in Fig. 4), which is slightly higher than the values determined by Seidl et al. [20]. The activation energy is comparable to the activation energy of 45 kJ/mol determined by Andersson and Inaba at 1.0 GPa from the slope in Fig. 3(a) [115]. Assuming an Avramov-type pressure dependence of T_g_ [126], [127] (see green solid line in Fig. 4) extrapolation of the volumetric high-pressure T_g_ data by Seidl et al. [20] would imply a 1 bar T_g_ of HDA of 115 ± 10 K. This is much lower than the T_g_ (HDA, 1 bar) ~ 140 K obtained by Mishima by linear extrapolation from data on emulsified salt solutions (red dashed line in Fig. 4). This difference is particularly noteworthy since the highest temperatures at which HDA can be retained at ambient pressure was found to be 125 K by Nelmes et al. [11], 134 K by Winkel et al. [86] and 136 K by Handle et al. [90]. Above this temperature a sharp volume increase of about 25% and the polyamorphic transformation to LDA are observed. One thus may speculate that this implies that the high-density liquid could be accessible even at ambient pressure in HDA samples, at least when studying well-relaxed samples showing high thermal stability.

### 3.2 Experiments at ambient pressure

For this reason highly thermally stable HDA samples were prepared following the approach of Winkel et al. [12], [86] and investigated using differential scanning calorimetry and dielectric relaxation spectroscopy [44]. The thermograms indeed show an increase in heat capacity at 116 K (see Fig. 1(b)) for a heating rate of 10 K/min. The increase in heat capacity is repeatable in cooling/heating cycles when taking care not to heat the sample above the temperature required for conversion to LDA. This is demonstrated in Fig. 1(b) by a sevenfold repetition of the measurement. Clearly, the curves superimpose perfectly (and could not be recognized as seven different thermograms if they were not shifted for clarity), and so necessarily the glass transition is not only repeatable, but also reversible and does not show signs of irreversible relaxation. The value of 116 K ( in Fig. 4) is in accord with expectations from an Avramov-type extrapolation of the pressure dependence of the volumetric T_g_ (see green solid line in Fig. 4). The increase in heat capacity ∆C_p_ at T_g_ was found to be between 3.6 J K^− 1^ mol^− 1^ (see Fig. 1(b)) and 4.8 J K^− 1^ mol^− 1^ [44]. This is about 4–5 times the value found previously for LDA's glass transition [41] and about the same as the heat capacity increase found at 1.0 GPa (see Fig. 1(a)) [117]. Compared to the ∆C_p_ observed in other glass-forming materials water values are rather small, but comparable to those obtained in strong liquids [128], [129]. Judging from the ∆C_p_'s one would conclude that HDL is a strong liquid, and low-density liquid water (LDL) an even stronger liquid, if not the strongest liquid known today [130]. The repeatability of the increase in heat capacity was checked by heating the sample several times into the glass transition region. In the experiment shown in Fig. 1(b) the sample was heated twice to 123 K, i.e., to slightly below the glass transition midpoint, and four more times to 128 K, i.e., halfway between glass transition midpoint and overshoot point, at which conversion to LDA starts. In the seventh heating run the trace exactly follows the trace obtained in all preceding heating runs. That is, the sample is in the same state even before the seventh heating run, which demonstrates the repeatability and the reversibility required for a glass transition. This conclusion was cross-checked by studying the dielectric relaxation of the quench-recovered, powdered amorphous ice near the calorimetric T_g_. Using broadband dielectric relaxation spectroscopy performed at ambient pressure frequency dependent dielectric loss peaks could be resolved in the 120–124 K range for HDL at 1 bar (see Fig. 2(b)), which is similar to the one observed by Andersson at 1.0 GPa (see Fig. 2(a)). Also for LDL audio-frequency dielectric loss peaks could be resolved at 140–151 K. From the observed peak frequencies and exploiting time temperature superposition a relaxation map could be constructed for both HDL and LDL (see Fig. 3(b)). The criterion of a dielectric relaxation time of 100 s yields dielectric glass transition temperatures at ambient pressure as 110 K for HDA ( in Fig. 4) and 126 K for LDA. The value deduced for VHDA at 1.0 GPa (see Fig. 3(a)) using this criterion is 122 K ( in Fig. 4). The relaxation times of both HDA/HDL and LDA/LDL follow an Arrhenius law. In the case of LDA/LDL a steepness index [125], [131] m ~ 14 can be extracted from the data, which justifies to call it a superstrong liquid [130]. Its superstrong nature was explained by the importance of quantum effects near the glass transition temperature [132], [133]. In the case of HDA/HDL the Arrhenius fit is of poorer quality, but instead a slight curvature can be spotted in the relaxation map. A steepness index m = 20–25 is derived from the data, which puts HDL in the category of strong liquids, in accord with the calorimetry result.

The calorimetry and dielectric relaxation spectroscopy data are consistent with a liquid nature of water above its T_g_ as originally suggested by Mishima and Suzuki [101]. The increase in heat capacity and the dielectric loss peak above T_g_ might, however, also be interpreted in terms of other scenarios such as an onset of proton mobility and rotational dynamics of water molecules, but no translation mobility as suggested by Fisher and Devlin [91]. Nevertheless, such an orientational glass transition seems unlikely: (i) the increase in heat capacity at the orientational glass transitions of ice I_h_, ice XII or ice V was found to be ≤ 1 J K^− 1^ mol^− 1^ [70], [93], [134], [135]. This is only about one fifth of the ∆C_p_ observed for HDA, in spite of a very similar local packing, (ii) introduction of additional point defects in the form of Bjerrum defects and ionic defects by doping with KOH does not alter the dynamics (see discussion above) and (iii) the dielectric loss of HDL resembles the pattern of supercooled liquids such as glycerol, showing an excess wing (see Fig. 2(b)), at variance with the mono-dispersive (Debye-like) pattern observed for ice crystals [136], [137] where proton dynamics governs the relaxation process. Of course, all of this represents circumstantial evidence, and direct measurements regarding a potential mechanical softening and a decrease in viscosity upon heating HDA near the glass transition would be desirable to clarify the nature of water's second glass transition pertaining to the high-density liquid.

The summary of glass transition temperatures for (V)HDA in Fig. 4 allows for two main conclusions: (i) the data can be interpreted within a coherent picture if distinct glass transition temperatures for HDA and VHDA are assumed. This corroborates the prior assertion that HDA and VHDA need to be regarded as two different polyamorphic states [10], [54]. (ii) The glass transition temperature for HDA increases with pressure. The slope is > 100 K/GPa between ambient pressure and 0.1 GPa (by comparing ambient pressure methods with high pressure methods) and ~ 40 K/GPa between 0.1 and 0.3 GPa. In addition, recent work clearly shows that HDA and LDA show two distinct glass transition temperatures, separated by 20 K [44]. That is, three glass transition temperatures need to be distinguished. While there has been progress in locating the HDA glass transition line in the pressure–temperature phase diagram, the line still needs to be defined for LDA and VHDA. For the former, just the point at 136 K at 1 bar is known, and for the latter from Andersson and Inaba's work it seems that the glass transition temperature is surprisingly low — much lower than the glass transition temperatures of HDA extrapolated to higher pressures (see green solid line and blue dotted line in Fig. 4).
